# Muscle-enriched microRNA miR-486 decreases in circulation in response to exercise in young men

**DOI:** 10.3389/fphys.2013.00080

**Published:** 2013-04-11

**Authors:** Wataru Aoi, Hiroyuki Ichikawa, Keitaro Mune, Yuko Tanimura, Katsura Mizushima, Yuji Naito, Toshikazu Yoshikawa

**Affiliations:** ^1^Laboratory of Health Science, Kyoto Prefectural UniversityKyoto, Japan; ^2^Department of Molecular Gastroenterology and Hepatology, Kyoto Prefectural University of MedicineKyoto, Japan

**Keywords:** exercise, maximum oxygen uptake, metabolism, microRNA, skeletal muscle

## Abstract

**Background:** MicroRNAs (miRNAs) are small non-coding RNAs involved in post-transcriptional gene regulation. miRNAs are taken in by intracellular exosomes, secreted into circulation, and taken up by other cells, where they regulate cellular functions. We hypothesized that muscle-enriched miRNAs existing in circulation mediate beneficial metabolic responses induced by exercise. To test this hypothesis, we measured changes in muscle-enriched circulating miRNAs (c-miRNAs) in response to acute and chronic aerobic exercise.

**Methods:** Eleven healthy young men (age, 21.5 ± 4.5 y; height, 168.6 ± 5.3 cm; and body weight, 62.5 ± 9.0 kg) performed a single bout of steady-state cycling exercise at 70% VO_2max_ for 60 min (acute exercise) and cycling training 3 days per week for 4 weeks (chronic exercise). Blood samples were collected from the antecubital vein before and after acute and chronic exercise. RNA was extracted from serum, and the levels of muscle-enriched miRNAs (miR-1, miR-133a, miR-133b, miR-206, miR-208b, miR-486, and miR-499) were measured.

**Results:** All of these miRNAs, except for miR-486, were found at too low copy numbers at baseline to be compared. miR-486 was significantly decreased by both acute (*P* = 0.013) and chronic exercise (*P* = 0.014). In addition, the change ratio of miR-486 due to acute exercise showed a significant negative correlation with VO_2max_ for each subject (*R* = 0.58, *P* = 0.038).

**Conclusion:** The reduction in circulating miR-486 may be associated with metabolic changes during exercise and adaptation induced by training.

## Introduction

MicroRNAs (miRNAs) are small non-coding RNAs of ~19–22 nucleotides in length; miRNAs regulate gene expression at the post-transcriptional level through translational inhibition or mRNA degradation (Ambros, 2004; Bartel, 2004). miRNA-mediated gene regulation is a key mechanism of post-transcriptional regulation in a wide range of physiological and pathological processes.

Recently, growing evidence has shown that some miRNAs are taken into intracellular vesicles (e.g., exosomes) and released into circulation without being degraded by RNase (Valadi et al., 2007). In addition, circulating miRNAs (c-miRNAs) can move from circulation into other cells and regulate their functions (Valadi et al., 2007). Thus, miRNAs are considered to be useful biomarkers, which could determine various interactions between tissues and also reflect physiological and pathological states. In fact, circulating levels of several miRNAs are changed in some types of cancer and cardiovascular diseases. In some cases, they reflect incidence risk and disease development. Indeed, c-miRNAs may contribute to the pathogenesis of the disease by regulating protein expression of target cells (Mitchell et al., 2008; Wang et al., 2009; Heneghan et al., 2010; Wang et al., 2010).

Several miRNAs act as modulators of muscle cell function such as proliferation, differentiation, hypertrophy, and nutrient metabolism (Chen et al., 2006; Cardinali et al., 2009; McCarthy et al., 2009; Dey et al., 2011). Some of these miRNAs are highly enriched in muscle tissue and hence often referred to as myomiRs. Four of these myomiRs, namely, miR-1, miR-133a/b, and miR-206, make up nearly 25% of miRNA expression in skeletal muscle in both humans and mice (Sempere et al., 2004; McCarthy, 2008). Other miRNAs, namely, miR-486, miR-208, and miR-499, are also encoded by muscle-specific genes such as *ankyrin* and *myosin heavy chain* (McCarthy et al., 2009; Small et al., 2010). Exercise and immobilization can change the level of miRNAs in skeletal muscle, which is suggested to account for phenotypic changes (Safdar et al., 2009; Aoi et al., 2010; Nielsen et al., 2010). In addition, they may also regulate other tissues by secretion to circulation. Indeed, some muscle-enriched miRNAs were detected in plasma and found to respond to pathological status in animal studies (Eisenberg et al., 2007; McCarthy et al., 2007; Deng et al., 2011; Mizuno et al., 2011).

Regular exercise can improve skeletal muscle function, including nutrient metabolism and muscle strength along with reducing the risk of cardiovascular disease, type 2 diabetes, and cancer. In addition to the adaptive effects by regular exercise, even a single bout of exercise induces various benefits including metabolic improvement. Although the detailed mechanism remains unknown, exercise-induced benefits affect not only skeletal muscle but also other tissues in the body. We hypothesized that c-miRNAs secreted from muscle tissue, or taken up by muscles or other organs, can mediate transient and adaptive responses to exercise. To our knowledge, it is not known if levels of muscle-enriched c-miRNAs are changed in response to exercise. To test this hypothesis, we investigated the levels of skeletal muscle-enriched miRNAs in circulation in response to metabolic changes induced by acute and chronic aerobic exercise in young sedentary male subjects. Here, we report that both acute and chronic exercise decrease the circulating levels of the muscle-enriched miRNA, miR-486.

## Methods

### Ethical approval and subjects

Eleven healthy young male subjects (age, 21.5 ± 4.5 y; height, 168.6 ± 5.3 cm; and body weight, 62.5 ± 8.5 kg) who were not habituated to a regular exercise regimen were recruited to participate in this study, which was approved by the ethics committee of Kyoto Prefectural University. All of the subjects signed a consent form after reading information about the study and having the procedures explained to them. One man retired before the completion of exercise training program because of deconditioning unrelated to the study. Therefore, data obtained from 10 subjects were analyzed in the training intervention. None of the subjects had any current or prior chronic disease, history of smoking, or current use of any medications. The characteristics of the subjects are shown in Table 1.

**Table 1:** **Subject characteristics and blood metabolic factors**.

	**Baseline**	**Post-exercise**	**Post-training**
Body weight (kg)	62.5 ± 9.0		61.8 ± 8.9^*^
Body mass index (kg/m^2^)	22.0 ± 3.0		21.7 ± 2.8^*^
VO_2max_ (ml/min/kg)	41.5 ± 7.9		46.1 ± 9.7^**^
Work load (watt)	201 ± 26		226 ± 31^**^
Blood glucose (mg/dl)	84 ± 10	87 ± 16	84 ± 6
Serum insulin (μU/ml)	11.9 ± 6.1	9.3 ± 9.8	12.0 ± 6.4
Blood lactate (mM)	1.0 ± 0.2	3.0 ± 1.4^**^	

Values represent as the mean ± standard deviation obtained from 10–11 subjects.

* P < 0.05 vs. baseline.

** P < 0.01 vs. baseline.

### Study design

Before the experiment, an incremental exercise test was performed to determine the maximum oxygen uptake (VO_2max_) of each subject using indirect calorimetry (Aeromonitor AE310S; Minato, Osaka, Japan) on a cycling ergometer (75XLII; Combi, Tokyo, Japan). The work load was gradually increased by 20 W every 2 min until oxygen consumption following 2 min of unloaded pedaling could not be increased further. Objective criteria for maximal effort included at least two of the following: (1) increased workload without corresponding increase in VO_2_; (2) respiratory exchange quotient equal to or greater than 1.10; and (3) a pedal cadence lower than 50 rpm in spite of maximal voluntary effort. The work-load at 70% VO_2max_ was estimated for each subject. As a result, all subjects were corresponded to volitional fatigue as at least one criteria.

A single-bout exercise experiment was performed 3 days following the incremental exercise test to eliminate the effect of transient metabolic changes that continue for a while in post-exercise. The subjects were asked not to eat or drink anything except for water from 22:00 to the next morning. On the experiment day, all subjects consumed 200 g of boiled rice as breakfast 2 h before each exercise session to normalize the effects of a pre-exercise meal. All participants performed a single bout of steady-state cycling exercise at 70% VO_2max_ for 60 min. Blood samples were collected from the antecubital vein before and after exercise, and blood glucose and lactate were measured using simple measuring instruments (Lactate Pro, GluTest; Arkray, Inc., Kyoto, Japan). Exercise training program was started within 2 weeks after the single-bout exercise. Body weight and blood pressure were measured to examine baseline condition level. Participants exercised at 70% VO_2max_ for 30 min three times per week for 4 weeks. Forty-eight hours after the last exercise session, blood samples were collected described above, and blood glucose was measured. VO_2max_, body weight, and blood pressure were measured again after 4 weeks of training.

Each blood sample was collected in a spit containing serum-separation agent, allowed to clot, and then centrifuged at 3500 rpm for 15 min at 4°C to separate serum. The isolated serum was used for the measurement of insulin (FALCO), and the remaining samples were immediately frozen at −80°C for miRNA analyses. The post-exercise change in plasma volume by a single bout of exercise was determined from hemoglobin and hematocrit concentrations, which were calculated using Dill and Costill's formula (Dill and Costill, 1974).

### Isolation of miRNAs and RT–PCR

Total RNA extraction was performed using the TRIzol LS reagent (Invitrogen, Carlsbad, CA). RT–PCR was performed using the RNA samples obtained from serum. We used ready-made solutions containing the primers and probes for human miR-1, miR-16, miR-133a, miR-133b, miR-206, miR-208b, miR-486, and miR-499 (Applied Biosystems, Foster City, CA), and performed real-time RT–PCR using an ABI 7300 system (Applied Biosystems). The *Caenorhabditis elegans* miRNA miR-39, which lacks sequence homology to human miRNAs, was used as a spiked control. Two femtomole of a chemically synthesized cel-miR-39 (Sigma-Aldrich Japan, Tokyo, Japan) was spiked into each serum sample (50 μl) after the addition of phenol solution to inhibit RNase. In addition, miR-16, a representative miRNA enriched in blood, was used as an endogenous control. The ratio of the signal for miRNAs to that for endogenous (miR-16) and exogenous (cel-miR-39) controls was calculated for each serum sample.

### Statistical analyses

All data are reported as the mean ± standard deviation. Because normal distribution was not obtained for all parameters, non-parametric analysis was used. Differences between pre- and post- experiment parameters were evaluated by Wilcoxon signed-rank test. Correlation between VO_2max_ and change ratio of miR-486 between before and after a single bout of exercise was evaluated by Spearman's correlation analysis. Statistical significance was accepted at the 5% level.

## Results

### Changes in subject characteristics in response to acute and chronic exercise

After 4 weeks of training, body weight (62.5 ± 9.0 kg to 61.8 ± 8.9 kg, *P* < 0.05) and body mass index (22.0 ± 3.0 kg/m^2^ to 21.7 ± 2.8 kg/m^2^, *P* < 0.05) were significantly decreased, and VO_2max_ (41.5 ± 7.9 ml/min/kg to 46.1 ± 9.7 ml/min/kg, *P* < 0.01) and work load (201 ± 26 watt to 226 ± 31 watt, *P* < 0.01) were significantly increased, compared with baseline levels (Table 1). The concentrations of blood glucose and serum insulin were not changed by either a single bout of exercise or 4-weeks training. The concentration of blood lactate was markedly increased by a single bout of exercise compared with baseline (1.0 ± 0.2 mM to 3.0 ± 1.4 mM, *P* < 0.01).

### Detection of muscle-specific miRNAs in serum

**Figure 1:**
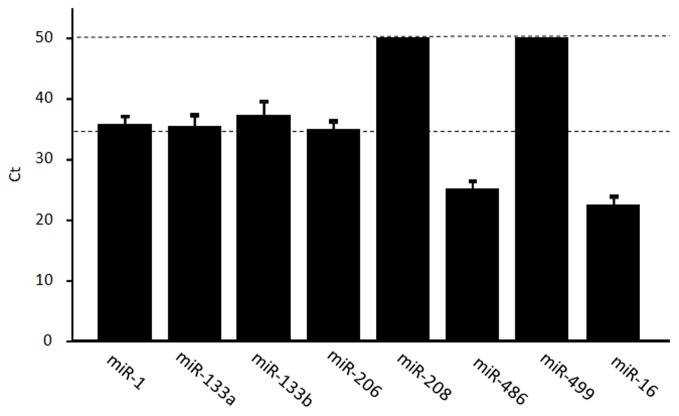
**Levels of muscle-enriched miRNAs in serum.** The levels of muscle-enriched miRNAs (miR-1, miR-133a, miR-133b, miR-206, miR-208b, miR-486, and miR-499) and miR-16 in serum were measured using real-time RT–PCR. miRNAs levels are expressed as a threshold cycle (Ct) detection. Values represent the mean ± standard deviation obtained from 11 subjects.

The miRNAs miR-1, miR-133a, miR-133b, miR-206, miR-208b, miR-486, and miR-499 are enriched in muscles, but typically found at low levels in other tissues. We examined the levels of muscle-specific miRNAs in circulation using real-time RT–PCR. For each individual sample, the mean expression value was calculated based on reference miRNAs according to a threshold cycle (Ct) value. The mean Ct value was 35.7 (miR-1), 35.4 (miR-133a), 35.2 (miR-133b), 34.9 (miR-206), and 25.1 (miR-486) (Figure 1). miR-208b and miR-499 could not be detected (>50 Ct) in five and six subjects, respectively, and were difficult to detect (>40 Ct) in the remaining subjects. The cut-off value for low-copy (<250 copies) c-miRNAs is typically 35 PCR cycles; below this value, miRNAs are difficult to quantify and accurately compare (Mestdagh et al., 2009; Agueli et al., 2010). Therefore, we judged that miRNAs except for miR-486 are difficult to be compared changes in response to exercise.

### Changes of circulating miR-486 in response to acute and chronic exercise

**Figure 2:**
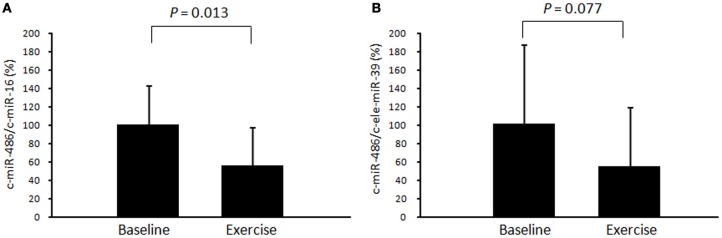
**Changes in circulating miR-486 in response to acute exercise.** The level of circulating miR-486 in response to a single bout of exercise was determined. The ratio of the signal for miR-486 to that for miR-16 **(A)** and cel-miR-39 **(B)** was calculated for each serum sample. Values represent the mean ± standard deviation obtained from 11 subjects.

**Figure A1:**
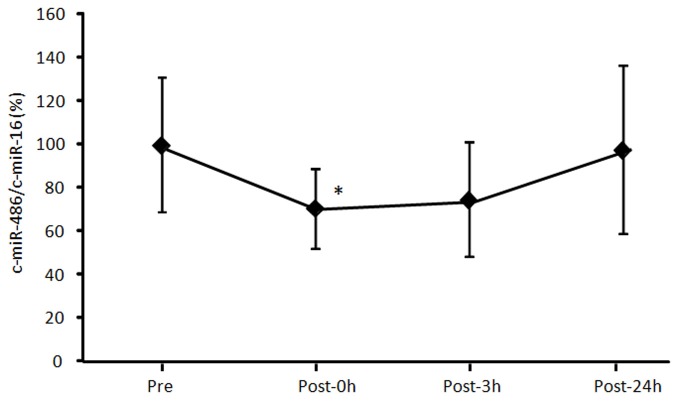
**Time-course change of circulating miR-486 in response to acute exercise.** The level of circulating miR-486 was determined before (Pre) and immediately (Post-0 h), 3 h (Post-3 h), 24 h (Post-24 h) after a single bout of exercise. The ratio of the signal for miR-486 to that for miR-16 was calculated for each serum sample. Values represent as the mean ± standard deviation obtained from six subjects. ^*^P < 0.05 vs. Pre.

**Figure A2:**
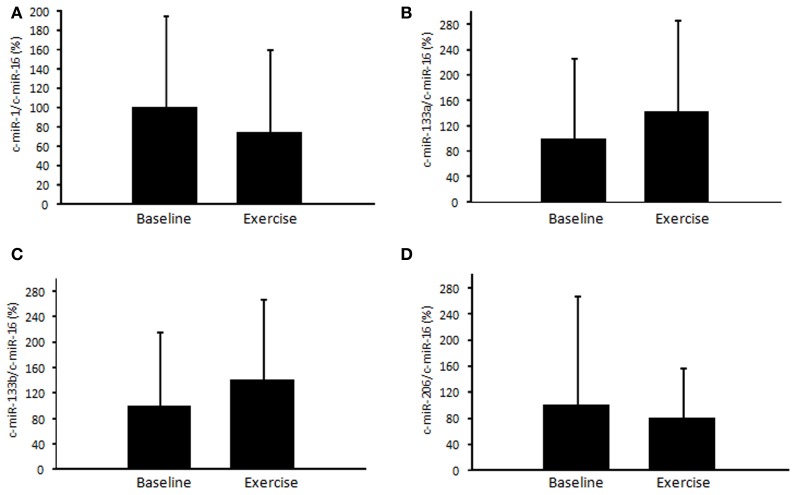
**Changes of circulating miRNAs in response to acute exercise.** The level of circulating miR-1 **(A)**, miR-133a **(B)**, miR-133b **(C)**, and miR-206 **(D)** in response to a single bout of exercise was determined. The ratio of the signal for microRNAs to that for miR-16 was calculated for each serum sample. Values represent as the mean ± standard deviation obtained from 11 subjects.

First, we examined time-course changes in circulating miR-486 after acute exercise. Samples from six subjects were obtained before and immediately, 3 h, and 24 h after exercise. Levels of circulating miR-486 were significantly reduced immediately after exercise but returned to baseline levels 24 h later (Supplemental Figure A1). Based on these findings, all of the following comparisons were performed between values before and immediately after exercise. We found that the level of c-miR-486 was significantly decreased after a single bout of exercise compared with baseline when the values were normalized to c-miR-16 (100 ± 43% to 56 ± 41%, *P* = 0.013) (Figure 2A). A similar tendency was also found when the values were normalized to c-ele-miR-39 (100 ± 87% to 54 ± 65%, *P* = 0.077) (Figure 2B). These findings showed that the reduction in c-miR-486 induced by acute exercise was recovered after 3 h and returned to baseline levels after 24 h (Figure A1). On the other hand, levels of miR-1, miR-133a, miR-133b, and miR-206 were not significantly changed by acute exercise (Figure A2).

**Figure 3:**
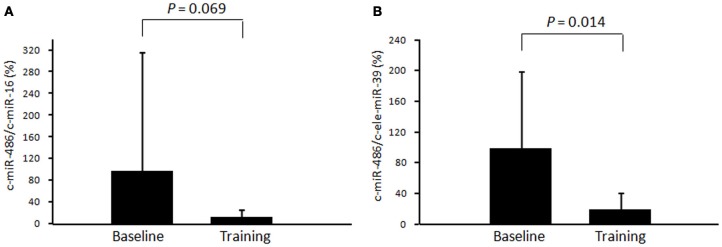
**Changes in circulating miR-486 in response to chronic exercise.** The level of circulating miR-486 in response to 4 weeks of exercise training was determined. The ratio of the signal for miR-486 to that for miR-16 **(A)** and cel-miR-39 **(B)** was calculated for each serum sample. Values represent the mean ± standard deviation obtained from 10 subjects.

The level of c-miR-486 (normalized to c-ele-miR-39) was significantly decreased after chronic exercise as compared to that before training (100 ± 99% to 20 ± 21%, *P* = 0.014) (Figure 3B). A similar tendency was also found when the values were normalized to c-miR-16 (100 ± 221% to 16 ± 14%, *P* = 0.069) (Figure 3A).

### Correlation between circulating miR-486 and VO_2max_

**Figure 4:**
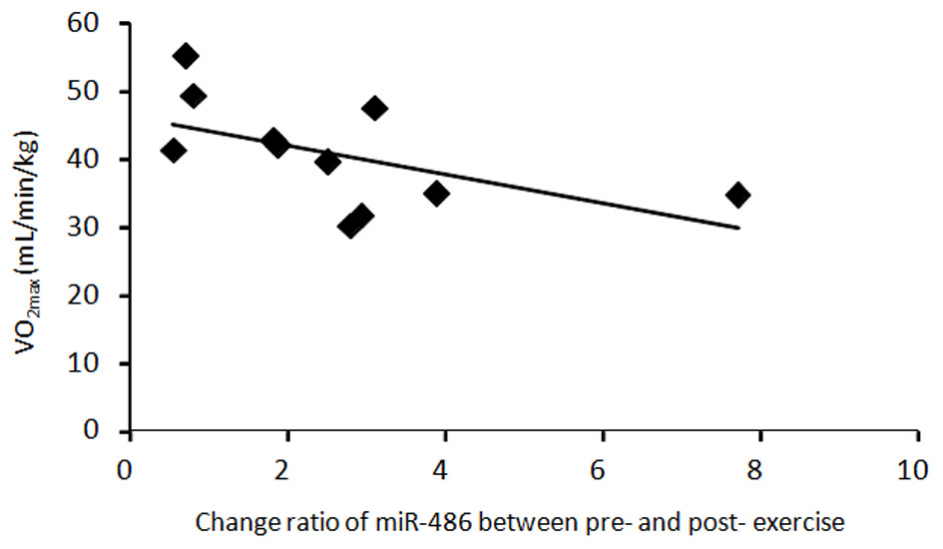
**The relationship between c-miR-486 and VO_2max_.** Change ratio of miR-486 between before and after a single bout of exercise and VO_2max_ values were plotted for each of 11 subjects. There was a significant negative correlation between the change ratio of circulating miR-486 (normalized to miR-16) after acute exercise and VO_2max_ (*R* = 0.58, *P* = 0.038).

There was a significant negative correlation between the change ratio of c-miR-486 (normalized to c-miR-16) after a single bout of exercise and VO_2max_ (*R* = 0.58, *P* = 0.038) (Figure 4). On the other hand, there was a tendency toward positive correlation between the change ratios of c-miR-486 (normalized to c-ele-miR-39) and serum insulin after training (*R* = 0.43, *P* = 0.107). The change ratio of c-miR-486 between pre- and post-acute and chronic exercise was not significantly correlated with the changes in body composition or blood glucose (data not shown).

## Discussion

Exercise affects the levels of circulating anti-angiogenic miRNAs, namely, miR-20a, miR-210, miR-221, miR-222, and miR-328 (Baggish et al., 2011). Several other miRNAs have also been reported to regulate skeletal muscle function. However, they are present in skeletal muscle at low levels, or are not muscle-specific. In the present study, we focused our attention on seven muscle-specific miRNAs. We observed that muscle-specific miRNAs were present at very low levels in serum, suggesting their low secretion from muscle cells into circulation. Therefore, it is difficult to consider the effects of these miRNAs on other tissues. The only exception was miR-486, which was found in serum at relatively higher levels. The levels of this miRNA are 10–20-times higher in skeletal and cardiac muscle cells than in other tissue cells (Small et al., 2010). Consistent with our results, several previous studies (Wang et al., 2010; Zampetaki et al., 2010; Konishi et al., 2012) have shown that miR-486 circulates in the blood at relatively high levels.

We found that the level of c-miR-486 is markedly decreased after both acute and chronic exercise compared to baseline. Several mechanisms may be responsible for the decrease in c-miR-486 in response to exercise. One mechanism may be reduction in secretion from muscle cells to circulation. Although the half-life of miR-486 is unclear, 60 min of exercise is probably too short a period to reflect a decrease in circulating levels by decrease of secretion. Another possibility is that degradation of c-miR-486 is accelerated by exercise. Stress induced by exercise, e.g., oxidative, hormonal, mechanical, and osmotic stress, may destroy exosome vehicles, leading to degradation of miRNAs by RNases. However, the levels of a control miRNA (c-miR-16) were not changed by either acute or chronic exercise, suggesting that significant degradation of exosomes did not occurred by exercise. Furthermore, exercise may accelerate uptake of c-miR-486 from circulation into certain recipient cells. Several studies (Valadi et al., 2007; Kosaka et al., 2010; Mittelbrunn et al., 2011) have shown through *in vitro* experiments that miRNAs contained in exosomes are transferable from outer media into recipient cells, where they affect their biological functions. Previously, we found that miR-486 level tended to increase in skeletal muscle after exercise training in mice (data not shown); this may partly be caused by uptake from circulation to muscle cells.

We observed a negative correlation between the change ratio of c-miR-486 by a single bout of exercise and VO_2max_ at baseline, which suggests that the reduction in c-miR-486 is associated with energy metabolism during exercise. Small et al. (2010) demonstrated that a major putative target of miR-486 is phosphatase and tensin homolog (PTEN), which is a negative regulator of phosphoinositide-3-kinase/Akt signaling, a major pathway downstream of the insulin receptor. Thus, miR-486 can regulate insulin-dependent glucose uptake in metabolic tissues such as the skeletal muscle. For maintaining muscle contraction during exercise, it is important to supply glucose as a major energy substrate. If substrate supply from blood glucose or muscle glycogen is not facilitated, muscle contraction is hampered. Subjects with low endurance capacity exhibit increased glucose uptake from blood into muscle cells because they have low glycogen contents in the muscle compared with those in subjects with high endurance capacity. A single bout of exercise activates insulin signaling in muscle cells during exercise, and this activation persists even after exercise. The miR-486 may conduct glucose uptake via activation of insulin signaling and suppression of PTEN, which contributes to maintenance of muscle contraction during exercise, especially in low-endurance subjects. In contrast, high endurance subjects may maintain glucose metabolism even if c-miR-486 is not taken into contracting muscle cells. Compared with subjects who have high endurance capacity, subjects who have high endurance capacity are better at lipid utilization than glucose utilization as an energy substrate during exercise at moderate intensity (Coggan et al., 1995); this may result from uptake of c-miR486 from blood into muscle cells. If so, it may be rather important to maintain the level of miR-486 in circulation during exercise.

We also found that 4 weeks of exercise training markedly reduced the level of c-miR486. Time-course analysis showed that the reduction in c-miR-486 induced by acute exercise was gradually recovered and returned to baseline levels 24 h after exercise; this suggests that the reduction after 4-weeks training was not affected by a final exercise bout. Although several mechanisms are likely involved in the adaptive reduction via chronic exercise, uptake from circulation into muscle cells may be similar to that in acute exercise because the level of miR-486 in skeletal muscle increased after 4 weeks of exercise training in our previous study (data not shown). Numerous studies (Perseghin et al., 1996; Goodyear and Kahn, 1998; Ross et al., 2000; Donnelly et al., 2003; Jakicic et al., 2003) have shown that exercise training adaptively improves insulin-dependent glucose uptake in skeletal muscle at the resting state. The miR-486 taken into muscles may partly contribute to metabolic improvement via translational suppression or mRNA degradation of PTEN. Indeed, exercise training can decrease expression of PTEN (Liu et al., 2012). We observed a tendency toward positive correlation between the reductions in c-miR-486 and insulin after training; this supports our hypothesis. In this case, the reduction in miR-486 in circulation may be considered a biomarker that reflects beneficial adaptation achieved by exercise training.

In conclusion, we found that the muscle-enriched miRNA, miR-486, is found in circulation in young healthy men. Circulating miR-486 was markedly reduced by acute and chronic exercise. There was a negative correlation between change ratio of miR-486 due to acute exercise and VO_2max_. These findings suggest that circulating miR-486 mediates metabolic changes during exercise and adaptation induced by training. It is unknown whether uptake of circulating miR-486 into muscle cells is accelerated by muscle contraction; if so, the mechanism of secretion and uptake remains to be elucidated. Further research is required to examine both the detailed mechanisms and the physiological relevance of the change in c-miR-486 in response to acute and chronic exercise.

### Conflict of interest statement

The authors declare that the research was conducted in the absence of any commercial or financial relationships that could be construed as a potential conflict of interest.

## References

[B1] Agueli C. Cammarata G. Salemi D. Dagnino L. Nicoletti R. La Rosa M. (2010). 14q32/miRNA clusters loss of heterozygosity in acute lymphoblastic leukemia is associated with up-regulation of BCL11a. Am. J. Hematol. 85, 575–578 10.1002/ajh.21758 20578197

[B2] Ambros V. (2004). The functions of animal microRNAs. Nature 431, 350–355 10.1038/nature02871 15372042

[B3] Aoi W. Naito Y. Mizushima K. Takanami Y. Kawai Y. Ichikawa H. (2010). The microRNA miR-696 regulates PGC-1{alpha} in mouse skeletal muscle in response to physical activity. Am. J. Physiol. Endocrinol. Metab. 298, E799–E806 10.1152/ajpendo.00448.2009 20086200

[B4] Baggish A. L. Hale A. Weiner R. B. Lewis G. D. Systrom D. Wang F. (2011). Dynamic regulation of circulating microRNA during acute exhaustive exercise and sustained aerobic exercise training. J. Physiol. 589, 3983–3994 10.1113/jphysiol.2011.213363 21690193 PMC3179997

[B5] Bartel D. P. (2004). MicroRNAs: genomics, biugenesis, mechanism and function. Cell 16, 281–297 14744438 10.1016/s0092-8674(04)00045-5

[B6] Cardinali B. Castellani L. Fasanaro P. Basso A. Alema S. Martelli F. (2009). Microrna-221 and microrna-222 modulate differentiation and maturation of skeletal muscle cells. PLoS ONE 4:e7607 10.1371/journal.pone.0007607 19859555 PMC2762614

[B7] Chen J. F. Mandel E. M. Thomson J. M. Wu Q. Callis T. E. Hammond S. M. (2006). The role of microRNA-1 and microRNA-133 in skeletal muscle proliferation and differentiation. Nat. Genet. 38, 228–233 10.1038/ng1725 16380711 PMC2538576

[B8] Coggan A. R. Swanson S. C. Mendenhall L. A. Habash D. L. Kien C. L. (1995). Effect of endurance training on hepatic glycogenolysis and gluconeogenesis during prolonged exercise in men. Am. J. Physiol. 268, E375–E383 7900783 10.1152/ajpendo.1995.268.3.E375

[B9] Deng Z. Chen J. F. Wang D. Z. (2011). Transgenic overexpression of miR-133a in skeletal muscle. BMC Musculoskelet. Disord. 12:115 10.1186/1471-2474-12-115 21615921 PMC3125252

[B10] Dey B. K. Gagan J. Dutta A. (2011). miR-206 and -486 induce myoblast differentiation by downregulating Pax7. Mol. Cell Biol. 31, 203–214 10.1128/MCB.01009-10 21041476 PMC3019853

[B11] Dill D. B. Costill D. L. (1974). Calculation of percentage changes in volumes of blood, plasma, and red cells in dehydration. J. Appl. Physiol. 37, 247–248 4850854 10.1152/jappl.1974.37.2.247

[B12] Donnelly J. E. Hill J. O. Jacobsen D. J. Potteiger J. Sullivan D. K. Johnson S. L. (2003). Effects of a 16-month randomized controlled exercise trial on body weight and composition in young, overweight men and women: the Midwest Exercise Trial. Arch. Intern. Med. 163, 1343–1350 10.1001/archinte.163.11.1343 12796071

[B13] Eisenberg I. Eran A. Nishino I. Moggio M. Lamperti C. Amato A. A. (2007). Distinctive patterns of microRNA expression in primary muscular disorders. Proc. Natl. Acad. Sci. U.S.A. 104, 17016–17021 10.1073/pnas.0708115104 17942673 PMC2040449

[B14] Goodyear L. J. Kahn B. B. (1998). Exercise, glucose transport, and insulin sensitivity. Annu. Rev. Med. 49, 235–261 10.1146/annurev.med.49.1.235 9509261

[B15] Heneghan H. M. Miller N. Lowery A. J. Sweeney K. J. Newell J. Kerin M. J. (2010). Circulating microRNAs as novel minimally invasive biomarkers for breast cancer. Ann. Surg. 251, 499–505 10.1097/SLA.0b013e3181cc939f 20134314

[B16] Jakicic J. M. Marcus B. H. Gallagher K. I. Napolitano M. Lang W. (2003). Effect of exercise duration and intensity on weight loss in overweight, sedentary women: a randomized trial. JAMA 290, 1323–1330 10.1001/jama.290.10.1323 12966123

[B17] Konishi H. Ichikawa D. Komatsu S. Shiozaki A. Tsujiura M. Takeshita H. (2012). Detection of gastric cancer-associated microRNAs on microRNA microarray comparing pre- and post-operative plasma. Br. J. Cancer 106, 740–747 10.1038/bjc.2011.588 22262318 PMC3322946

[B18] Kosaka N. Iguchi H. Yoshioka Y. Takeshita F. Matsuki Y. Ochiya T. (2010). Secretory mechanisms and intercellular transfer of microRNAs in living cells. J. Biol. Chem. 285, 17442–17445 10.1074/jbc.M110.107821 20353945 PMC2878508

[B19] Liu G. Detloff M. R. Miller K. N. Santi L. Houlé J. D. (2012). Exercise modulates microRNAs that affect the PTEN/mTOR pathway in rats after spinal cord injury. Exp. Neurol. 233, 447–456 10.1016/j.expneurol.2011.11.018 22123082 PMC3268901

[B20] McCarthy J. J. (2008). MicroRNA-206: the skeletal muscle-specific myomiR. Biochim. Biophys. Acta 1779, 682–691 10.1016/j.bbagrm.2008.03.001 18381085 PMC2656394

[B21] McCarthy J. J. Esser K. A. Andrade F. H. (2007). MicroRNA-206 is overexpressed in the diaphragm but not the hindlimb muscle of mdx mouse. Am. J. Physiol. Cell Physiol. 293, C451–C457 10.1152/ajpcell.00077.2007 17459947

[B22] McCarthy J. J. Esser K. A. Peterson C. A. Dupont-Versteegden E. E. (2009). Evidence of MyomiR network regulation of beta-myosin heavy chain gene expression during skeletal muscle atrophy. Physiol. Genomics 39, 219–226 10.1152/physiolgenomics.00042.2009 19690046 PMC2789671

[B23] Mestdagh P. Van Vlierberghe P. De Weer A. Muth D. Westermann F. Speleman F. (2009). A novel and universal method for microRNA RT-qPCR data normalization. Genome Biol. 10, R64 10.1186/gb-2009-10-6-r64 19531210 PMC2718498

[B24] Mitchell P. S. Parkin R. K. Kroh E. M. Fritz B. R. Wyman S. K. Pogosova-Agadjanyan E. L. (2008). Circulating microRNAs as stable blood-based markers for cancer detection. Proc. Natl. Acad. Sci. U.S.A. 105, 10513–10538 10.1073/pnas.0804549105 18663219 PMC2492472

[B25] Mittelbrunn M. Gutiérrez-Vázquez C. Villarroya-Beltri C. González S. Sánchez-Cabo F. González M. Á. (2011). Unidirectional transfer of microRNA-loaded exosomes from T cells to antigen-presenting cells. Nat. Commun. 2:282 10.1038/ncomms1285 21505438 PMC3104548

[B26] Mizuno H. Nakamura A. Aoki Y. Ito N. Kishi S. Yamamoto K. (2011). Identification of muscle-specific microRNAs in serum of muscular dystrophy animal models: promising novel blood-based markers for muscular dystrophy. PLoS ONE 6:e18388 10.1371/journal.pone.0018388 21479190 PMC3068182

[B27] Nielsen S. Scheele C. Yfanti C. Akerström T. Nielsen A. R. Pedersen B. K. (2010). Muscle specific microRNAs are regulated by endurance exercise in human skeletal muscle. J. Physiol. 588, 4029–4037 10.1113/jphysiol.2010.189860 20724368 PMC3000590

[B28] Perseghin G. Price T. B. Petersen K. F. Roden M. Cline G. W. Gerow K. (1996). Increased glucose transport-phosphorylation and muscle glycogen synthesis after exercise training in insulin-resistant subjects. N. Engl. J. Med. 335, 1357–1362 10.1056/NEJM199610313351804 8857019

[B29] Ross R. Dagnone D. Jones P. J. Smith H. Paddags A. Hudson R. (2000). Reduction in obesity and related comorbid conditions after diet-induced weight loss or exercise-induced weight loss in men. A randomized, controlled trial. Ann. Intern. Med. 133, 92–103 10896648 10.7326/0003-4819-133-2-200007180-00008

[B30] Safdar A. Abadi A. Akhtar M. Hettinga B. P. Tarnopolsky M. A. (2009). miRNA in the regulation of skeletal muscle adaptation to acute endurance exercise in C57Bl/6J male mice. PLoS ONE 4:e5610 10.1371/journal.pone.0005610 19440340 PMC2680038

[B31] Sempere L. F. Freemantle S. Pitha-Rowe I. Moss E. Dmitrovsky E. Ambros V. (2004). Expression profiling of mammalian microRNAs uncovers a subset of brain-expressed microRNAs with possible roles in murine and human neuronal differentiation. Genome Biol. 5, R13 10.1186/gb-2004-5-3-r13 15003116 PMC395763

[B32] Small E. M. O'Rourke J. R. Moresi V. Sutherland L. B. McAnally J. Gerard R. D. (2010). Regulation of PI3-kinase/Akt signaling by muscle-enriched microRNA-486. Proc. Natl. Acad. Sci. U.S.A. 107, 4218–4223 10.1073/pnas.1000300107 20142475 PMC2840099

[B33] Valadi H. Ekström K. Bossios A. Sjöstrand M. Lee J. J. Lötvall J. O. (2007). Exosome-mediated transfer of mRNAs and microRNAs is a novel mechanism of genetic exchange between cells. Nat. Cell Biol. 9, 654–659 10.1038/ncb1596 17486113

[B34] Wang G. K. Zhu J. Q. Zhang J. T. Li Q. Li Y. He J. (2010). Circulating microRNA: a novel potential biomarker for early diagnosis of acute myocardial infarction in humans. Eur. Heart J. 31, 659–666 10.1093/eurheartj/ehq013 20159880

[B35] Wang K. Zhang S. Marzolf B. Troisch P. Brightman A. Hu Z. (2009). Circulating microRNAs, potential biomarkers for drug-induced liver injury. Proc. Natl. Acad. Sci. U.S.A. 106, 4402–4407 10.1073/pnas.0813371106 19246379 PMC2657429

[B36] Zampetaki A. Kiechl S. Drozdov I. Willeit P. Mayr U. Prokopi M. (2010). Plasma microRNA profiling reveals loss of endothelial miR-126 and other microRNAs in type 2 diabetes. Circ. Res. 107, 810–817 10.1161/CIRCRESAHA.110.226357 20651284

